# Implicit gender bias among US resident physicians

**DOI:** 10.1186/s12909-019-1818-1

**Published:** 2019-10-29

**Authors:** Matt Hansen, Amanda Schoonover, Barbara Skarica, Tabria Harrod, Nathan Bahr, Jeanne-Marie Guise

**Affiliations:** 1 Center for Policy and Research in Emergency Medicine, Oregon Health & Science University, CR114, 3181 SW Sam Jackson Pk Rd, Portland, OR 97239 USA; 2 Department of Obstetrics & Gynecology, Oregon Health & Science University, Portland, USA

**Keywords:** Implicit bias, Gender bias, Medical education, Graduate medical education, Leadership

## Abstract

**Background:**

The purpose of this study was to characterize implicit gender bias among residents in US Emergency Medicine and OB/GYN residencies.

**Methods:**

We conducted a survey of all allopathic Emergency Medicine and OB/GYN residency programs including questions about leadership as well as an implicit association test (IAT) for unconscious gender bias. We used descriptive statistics to analyze the Likert-type survey responses and used standard IAT analysis methods. We conducted univariate and multivariate analyses to identify factors that were associated with implicit bias. We conducted a subgroup analysis of study sites involved in a multi-site intervention study to determine if responses were different in this group.

**Results:**

Overall, 74% of the programs had at least one respondent. Out of 14,234 eligible, 1634 respondents completed the survey (11.5%). Of the five sites enrolled in the intervention study, 244 of 359 eligible residents completed the survey (68%). Male residents had a mean IAT score of 0.31 (SD 0.23) and females 0.14 (SD 0.24), both favoring males in leadership roles and the difference was statistically significant (*p* < 0.01). IAT scores did not differ by postgraduate year (PGY). Multivariable analysis of IAT score and participant demographics confirmed a significant association between female gender and lower IAT score. Explicit bias favoring males in leadership roles was associated with increased implicit bias favoring males in leadership roles (r = 0.1 *p* < 0.001).

**Conclusions:**

We found that gender bias is present among US residents favoring men in leadership positions, this bias differs between male and female residents, and is associated with discipline. Implicit bias did not differ across training years, and is associated with explicit bias.

## Background

The US healthcare system is widely reported to suffer from high rates of patient safety problems. Several studies have identified team leadership as an important factor in many patient safety problems, and the Joint Commission has reported that inadequate leadership is associated with half of serious injuries or unanticipated deaths in the healthcare system [1]. Implicit or unconscious biases are, by definition, not overtly perceived by individuals and affect behavior on an unconscious level and therefore may be challenging to change. Implicit gender-based biases are important to recognize since they may hinder an individual’s ability to perform tasks effectively and to lead others, which may increase risk of patient safety problems and suboptimal care to patients.

The Implicit Association Test (IAT) is commonly used to measure implicit bias and has been used in several domains including race, gender, weight, and age [2–5]. Previous studies have found implicit racial bias among US physicians, though there has been limited investigation of implicit gender bias among physicians [6–9].

In academic medicine, men are implicitly viewed as leaders more than women [10]. In a study of internal medicine residents, most felt that gender was among the top 3 disadvantages in directing a care team [11]. Female residents have described feeling stress when violating gender behavioral norms when leading a cardiac resuscitation [12]. Residents from a broad range of specialties have noted gender differences in how their communication is perceived. Female residents reported their decisions were challenged more frequently than males and also reported receiving negative feedback in residency evaluations for showing assertive leadership behaviors [13, 14].

Female residents have also reported that the attitudes of male supervisors have made it difficult to cope with medical errors [15]. Though implicit gender bias in medicine has the potential to have a profound impact on team leadership, communication, and gender representation in academic leadership positions, the prevalence and effect of implicit gender bias on the performance of medical teams and team leaders has not been explored.

The objective of this study is to use the IAT to estimate the magnitude of implicit gender bias among resident physicians and explore associations between implicit bias and gender, discipline, year in training, explicit bias, and confidence in leadership skills.

## Methods

### Participants

We administered a leadership survey and IAT to residents from the 165 Emergency Medicine and 229 Obstetrics & Gynecology residencies in the US. We included all residencies that included Obstetrics & Gynecology or Emergency Medicine training programs. This included a number of dual residencies in Emergency Medicine including Emergency Medicine/Internal Medicine, Emergency Medicine/Internal Medicine/Critical Care, and Emergency Medicine/Pediatrics. The IAT and survey were conducted in conjunction with another study (results to be reported elsewhere) that evaluated two leadership curricula among residents in five specific academic medical centers. In these sites, study staff directly contacted residents and sent periodic reminders by their program coordinator or director to complete the survey, and thus distinctly higher response rates were expected among this subgroup.

### Measures

This study incorporated a leadership survey of all US Emergency Medicine and Obstetrics & Gynecology residents. We chose these specialties because they have traditionally been female (OB/GYN) and male (EM) dominated. The leadership survey was adapted from the previously developed and tested International Center for Executive Leadership in Academics’ Leadership Learning and Career Development Survey (LLCD) [16]. The LLCD survey includes Likert-type questions that ask respondents to rate the importance of, and their confidence in, various leadership attributes. In addition to the LLDC questions, we added specific questions to the survey that related to leading teams in acute care medicine. The survey also included an IAT to identify implicit gender bias among the respondents. After initial drafting of the survey adaptation, we discussed the questions in a serious of “talk aloud” sessions among the multidisciplinary research team. The Institutional Review Board of Oregon Health & Science University approved this study (IRB Number IRB00011053). Each participant individually consented to participation using an electronic written consent process that was required to be completed before the IAT could be accessed.

### Implicit association test

Each survey included a link to an IAT that was modeled after a previously developed IAT specifically designed to assess gender bias in leadership [17, 18]. We used male and female names instead of using photographs which are often used in the IAT to reduce potential bias from physical appearance. The test was comprised of 7 sections (blocks): 5 practice blocks with 20 trials each and 2 test blocks with 40 trails each (Table 1). Only data gathered during test blocks are used to analyze implicit bias. The study participants were randomized into two groups with differing order of block appearance to reduce the effect that order may have on performance. Our research team selected the male and female names and we felt by consensus to best exemplify male and female genders in the US. The gender IAT incorporated in this study included the following categories and descriptors: leader (director, president, executive, chief, boss), helper (assistant, junior, subordinate, co-worker, employee), male (Erik, Paul, John, Michael, Robert, David), and female (Julia, Ann, Emily, Rebecca, Sarah, Mary). Participants sorted the male and female names, as well the descriptors for leaders and helpers into these categories using keystrokes on computer keyboard. The time that it took participants to answer, as well as whether the answer was correct or not were recorded and used in subsequent analyses. The IAT was pilot tested among study team members. We implemented the IAT algorithm online using JavaScript. The IAT was able to be run on any platform with a modern web browser, and sent back the results which then were stored in an SQL database [19].

**Table 1:** Task Sequence of the LEADS IAT (Greenwald 2003)

						Category Label	
Block	No. of Trials	Function	Task	Items assigned to left-key response	Items assigned to right-key response	Left key	Right key
Randomization Group 1: Task Sequence of the LEADS IAT (Greenwald 2003)							
1	20	Practice	Attribute discrimination	Helper roles	Leader roles	Helper	Leader
2	20	Practice	Target discrimination	Male names	Female names	Male	Female
3	20	Practice	Initial combined task (practice)	Male names + Helper roles	Female names + Leader roles	Male, Helper	Female, Leader
4	40	Test	Initial combined task (test)	Male names + Helper roles	Female names + Leader roles	Male, Helper	Female, Leader
5	20	Practice	Reversed attribute discrimination	Leader roles	Helper roles	Leader	Helper
6	20	Practice	Reversed combined task (practice)	Female names + Leader roles	Male names + Helper roles	Female, Leader	Male, Helper
7	40	Test	Reversed combined task (test)	Female names + Leader roles	Male names + Helper roles	Female, Leader	Male, Helper
Randomization Group 2: Task Sequence of the LEADS IAT (Greenwald 2003)							
1	20	Practice	Attribute discrimination	Leader roles	Helper roles	Leader	Helper
2	20	Practice	Target discrimination	Male names	Female names	Male	Female
3	20	Practice	Initial combined task (practice)	Female names + Leader roles	Male names + Helper roles	Female, Leader	Male, Helper
4	40	Test	Initial combined task (test)	Female names + Leader roles	Male names + Helper roles	Female, Leader	Male, Helper
5	20	Practice	Reversed attribute discrimination	Helper roles	Leader roles	Helper	Leader
6	20	Practice	Reversed combined task (practice)	Male names + Helper roles	Female names + Leader roles	Male, Helper	Female, Leader
7	40	Test	Reversed combined task (test)	Male names + Helper roles	Female names + Leader roles	Male, Helper	Female, Leader

The measure used to represent implicit bias in this study is called the IAT D-Score (IAT_D_). Greenwald et al. (1998) developed the IAT and initially used the difference in latencies of responses for the two test blocks as the implicit bias measure. In 2003, Greenwald et al. published new methodology for analyzing IAT results, and concluded that the D-score had the best performance on the criterion for implicit-explicit bias correlation, and was the second best measure for having low correlation with average latency [20, 21]. Latencies are the difference in time it took respondents to select the response from the time the prompt was presented [17, 21]. Negative values indicate bias favoring women as leaders, positive values indicate bias favoring men as leaders, and values closest to zero indicate no preference or implicit bias. SAS was used for all preparatory transformations of the IAT latencies and D-scores (Version 9.4, SAS Institute, Inc. Cary, NC).

### Survey content-demographics

Demographic information included the resident’s specialty, year of post-graduate training, US state, institution, gender, and race/ethnicity.

### Survey content-leadership attributes

**Fig. 1:**
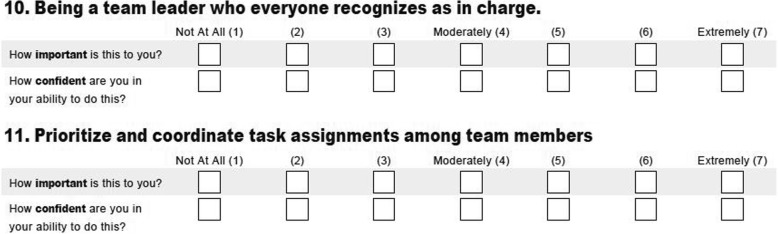
Example of leadership survey questions and survey format

Residents rated how important they viewed specific leadership attributes to be, followed by how confident they were in their abilities in these attributes (Fig. 1) on a 7-point Likert-type scale with 1 being not at all important/confident, 4 being moderately important/confident, and 7 being extremely important/confident. The questions in this portion related to effectiveness of leadership, being recognized as a leader, relating to and responding to team members, and situational awareness. Figure 1 displays an example of the format from this portion of the survey.

### Survey content-gender/sex and leadership

We used a Likert-type scale to ask respondents the degree to which they thought their own gender influenced team members’ response to them as leaders. The question was phrased, “Please indicate the degree to which you think (gender/sex) influences how team members respond to you as a Leader.” Possible answers included “extremely harmful”, “harmful”, “somewhat harmful”, “no effect”, “somewhat beneficial”, “beneficial”, and “extremely beneficial”. Finally, explicit attitudes toward gender were assessed by residents who rated whether men or women were more effective leaders on a 7-point scale with “men are extremely more effective leaders than women” on one end of the scale and “women are extremely more effective leaders than men” on the other.

### Survey administration

The survey was administered online via a customized website created for the project from April 2015 to February 2016. This website recorded both the IAT scores and survey responses for each individual. We partnered with the Council of Residency Directors (CORD) for Emergency Medicine and the Council on Residency Education in Obstetrics & Gynecology (CREOG) to deliver the survey. To distribute the study invitation we contacted residency directors and coordinators from each program and requested they forward a link with the survey to the residents in their programs. Prior to distribution of the survey, we sent a pre-letter via email to program directors and coordinators indicating we were going to send a survey. This message included endorsements from CORD and CREOG. Between 3 and 7 days after the pre-letter, we sent the survey to residency directors and coordinators requesting they forward the survey to their residents. After 7–10 days from the initial survey we sent a reminder and thank you note, then 7–10 days after that, we resent the links to the survey. We sent all communications through residency directors and coordinators.

### Analysis

We assessed differences in IAT_D_ scores among participants with different demographic characteristics including gender, race/ethnicity, year of training, and discipline using t tests for binary variables and Analysis of Variance (ANOVA) for variables with more than two categories. We used Pearson’s correlation to assess relationships between demographic characteristics.

To test whether gender and year in training were significant predictors of implicit bias (IAT_D_), we fit multiple linear regression models to the data. The fully adjusted model included sex, year in training, race/ethnicity, and discipline. We expected that implicit bias would be lower for residents who were more advanced in their training. We also included race as a covariate because we believed it could affect implicit bias. Lastly, discipline was included in the model because we expected that the differences in training modalities, department culture, and clinical experiences would affect implicit bias between OB/GEYN and EM residents. To test if discipline modified the relationship between gender and IATD, we stratified by discipline and included an interaction term in the model.

We evaluated explicit bias by directly asking residents if males and females were better leaderes and analyzed responses to this question by gender. Next, the explicit bias variable was divided into three categories: no explicit bias, bias favoring females as more effective leaders, and bias favoring males as more effective leaders. We assessed differences in mean IAT_D_ between these groups using ANOVA including Bonferroni corrections for multiple comparisons.

To estimate correlation between residents’ perception of how their own gender affects team members’ response to them as a leaders and implicit bias (IAT_D_), we used Pearson’s correlation coefficient. Frequency tables and ANOVA were used to explore how this response differed by gender.

Lastly, a measure of overall personal confidence in leadership was calculated by taking the average of residents’ responses to several key questions related to confidence in specific leadership skills. To explore the association between residents’ confidence in their leadership skills and implicit bias, we used Pearson’s correlation coefficient and linear regression adjusting for gender, race/ethnicity, year in training, and discipline.

To assess volunteer bias in the overall sample of all eligible U.S. residency programs, we completed a subset analysis of the residents from the five specific sites of the sub-study as the response rate was much higher in those programs (68%). All analyses were conducted with Stata (StataCorp. 2017. Stata Statistical Software: Release 15. College Station, TX: StataCorp LLC).

## Results

### Survey response rate

Overall, 74% of the programs had at least one respondent. In total, 1634 residents completed the survey from an estimated 14,243 eligible residents (11.5%). Out of the five sites enrolled in the randomized controlled trial, 244 of 359 (68%) eligible residents completed the survey and IAT.

### Demographics and overall implicit bias

Demographic characteristics including gender, race/ethnicity, year of training, and discipline are found in Table 2. Both male and female residents, on average, demonstrated implicit bias favoring men as leaders. Unadjusted IAT_D_ scores were significantly different between females and males, with females having a mean IAT_D_ of 0.14 and males having a mean IAT_D_ of 0.31 (t = − 14.4, *p*-value< 0.001). EM residents average IAT_D_ of 0.25 was found to be significantly higher than OB/GYN IAT_D_ mean of 0.16 (t = − 7.14, *p*-value< 0.001). When divided by sub-specialties within EM, sub-specialty was also associated with mean IAT scores (F = 19.2, *p*-value< 0.001). We did not find a significant difference in unadjusted IAT_D_ between “white” residents and “non-white” residents (t = − 0.79, *p*-value = 0.43), and year in training (F = 0.83, *p*-value = 0.51) (Table 2).

**Table 2:** Resident demographic characteristics and IAT_D_ scores

Demographic Characteristics	n (%)	mean IAT_D_	SD	t or F*	*p*-value
Overall	1634 (100)	0.20	0.25		
Gender					
Male	638 (39)	0.31	0.23	−14.4	< 0.001
Female	996 (61)	0.14	0.24		
Race/ethnicity					
White	1240 (76)	0.21	0.25	−0.79	0.43
Not white	394 (24)	0.19	0.26		
Year in training					
1	590 (36)	0.21	0.27	0.83*	0.51
2	436 (27)	0.21	0.23		
3	398 (24)	0.20	0.25		
4	196 (12)	0.18	0.25		
5+	14 (1)	0.22	0.19		
Discipline					
OB/GYN	786 (48)	0.16	0.24	19.2*	< 0.001
EM/EM + FM	825 (50)	0.25	0.25		
EM + IM	11 (1)	0.22	0.27		
EM + PEDS	12 (1)	0.07	0.22		
Discipline					
OB/GYM	786 (48)	0.16	0.24	−7.14	< 0.001
EM	848 (52)	0.25	0.25		

*Represents when an "F" statistic was used

### Gender and implicit bias

The results of the linear regression revealed that, on average, male residents’ IAT_D_ was 0.17 points greater than females, after adjusting for year in training, race/ethnicity, and specialty (95% CI: 0.15,0.20; *p*-value< 0.001) (Table 3). To assess the collinearity introduced into the model by the correlation between gender and specialty, we stratified the linear regression analysis by OB/GYN and EM. When stratified by specialty, the effect of gender on implicit bias differed between OB/GYN and EM residents, with the mean difference in IAT_D_ between females and males being 0.13 (95% CI: 0.08, 0.18) and 0.19 (95% CI: 0.16, 0.23), respectively (Table 3). Because of this difference in the gender-effect, we added an interaction term for gender and specialty to the linear regression model. The results show that the difference in IAT_D_ between females and males is magnified for emergency medicine residents (*p*-value = 0.04) (Table 3).

**Table 3:** Average IAT_D_ (95% CI)^a^ from final model with interaction term

		Sex	
		Female	Male
Discipline	OB/GYN	0.15 (0.11,0.19)	0.28 (0.22,0.33)
	EM	0.13 (0.09,0.17)	0.32 (0.28,0.36)
Key		Sex	
		Female	Male
Discipline	OB/GYN	B_0_	B_0_ + B_sex_
	EM	B_0_ + B_discipline_	B_0_ + B_sex_ + B_discipline_ + B_sex*discipline_

^a^adjusted for race/ethnicity and year in training

### Implicit and explicit bias

We directly asked participants if they felt men or women were more effective leaders on a 7-point scale. Frequency and distribution of the explicit bias measure are found in Table 4. In general, the large majority of both men and women reported that men and women were equally effective as leaders.

**Table 4:** Frequency and distribution of explicit bias measure

	Females		Males		Total	
	n	%	n	%	n	%
Men are extremely more effective leaders than women.	0	0.00	0	0.00	0	0.00
Men are somewhat more effective leaders than women.	14	1.41	14	2.19	28	1.71
Men are slightly more effective leaders than women.	94	9.44	57	8.93	151	9.24
Men and women are equally effective leaders.	826	82.9	558	87.5	1384	84.7
Women are slightly more effective leaders than men.	48	4.82	6	0.94	54	3.31
Women are somewhat more effective leaders than men.	14	1.41	2	0.31	16	0.98
Women are extremely more effective leaders than men.	0	0.00	1	0.16	1	0.06
Total	996		638		1634	

When condensed into three categories of explicit bias, (bias favoring men, neutral, and bias favoring women) residents who reported that men are more effective leaders had a mean IAT_D_ score of 0.26. Those who reported women as more effective leaders had a mean IAT_D_ score of 0.12, and those who reported that men and women are equally effective as leaders had a mean IAT_D_ score of 0.20 (Table 5). There was a significant association between the 3-category explicit bias measure and implicit bias (F = 8.03, *p-*value< 0.001).

**Table 5:** ANOVA assessing differences in mean IAT_D_ between three explicit bias groups

	n	Mean IAT_D_	F	*p*-value
Bias favoring men	71	0.26	8.03	< 0.001
Bias favoring women	179	0.12		
No bias	1384	0.2		

### Perception as leader and gender

Residents reported how they felt their own gender influenced team members’ perception of them as leaders. While 61% of males view their gender as being beneficial to how team members perceive them as leaders, only 7% of women felt that their gender was beneficial (Table 6). An approximately equal proportion of females and males reported that their gender has no effect on how they are perceived as leaders (39 and 37%, respectively). No males reported that their gender was extremely harmful or harmful, and only 1% of males said that their gender was somewhat harmful. On the other hand, 44% of females viewed their gender as somewhat harmful, 10% as harmful, and 1% as extremely harmful.

**Table 6:** Influence of gender on how resident is responded to as leader

	Females		Males		Total	
	n	%	n	%	n	%
Extremely Harmful	12	1	0	0	12	1
Harmful	97	10	2	0	99	6
Somewhat Harmful	442	44	9	1	451	28
No Effect	372	39	238	37	610	37
Somewhat Beneficial	52	5	185	29	237	15
Beneficial	14	1	175	28	189	12
Extremely Beneficial	6	1	28	4	34	2
Total	995		637		1632	

### Implicit bias and leadership confidence

We assessed the association between the IAT score and the residents’ responses to a subset of the leadership confidence questions we felt may be associated with implicit bias. We hypothesized that due to stereotypically assertive male leadership style, those with high confidence in their own leadership skills would have more bias towards men as leaders. We found that there was a small-magnitude but statistically significant correlation between implicit bias and confidence in leadership skills (r = 0.08, *p*-value = 0.002). When assessing the association between IAT_D_ and mean confidence in leadership skills, the unadjusted model showed that a 1-point increase in leadership confidence is associated with a 0.02 increase in implicit bias (β = 0.02, 95% CI: 0.02, 0.04). In addition, after adjusting for gender, race/ethnicity, year in training, and specialty, this association was no longer significant (β = 0.01, 95% CI: − 0.004, 0.03).

### Subgroup analysis of 5 sub-sites

Among residents from the sites participating in the sub-study, the response rate was much higher than the overall sample. We conducted a subset analysis of residents from those sites that to evaluate for potential bias in the larger sample with a much lower response rate. Overall results and trends were very similar between the RCT sites and all residency programs. In short, associations between implicit bias, gender, specialty explicit bias, and influence of gender were all similar in this subset of residents with higher response rates. There were some differences in statistical significance between the overall sample and the subset which were likely due to the smaller sample size resulting in lack of power to detect significant differences (*n* = 244).

## Discussion

To our knowledge, this is the first study to administer the gender/leadership IAT to resident physicians. We found that both men and women have implicit bias favoring men as leaders among residents in US Emergency Medicine and Obstetrics & Gynecology programs. This comes at a time when women have represented nearly half of students enrolled in US medical schools for over 20 years indicating the problem cannot be attributed to under-representation of women in medicine [22].

We found implicit bias differed significantly by gender, with males having greater implicit bias than females after adjusting for year in training, race/ethnicity, and specialty. We also found that the association between gender and implicit bias differed between Emergency Medicine residents, who are majority male, and Obstetrics and Gynecology residents, who are majority female, after controlling for year in training and race/ethnicity. The difference between males’ and females’ implicit bias was amplified among EM residents. This interaction between specialty and gender may be due to the fact that OB/GYN is predominately female-led, while EM is mostly male-led. Thus, male OB/GYN residents would be accustomed to women leadership and would exhibit less implicit bias favoring male leaders. We also found some correlation between implicit and explicit bias and implicit bias was highest among those who had explicit bias favoring male leaders. Male residents generally felt their gender resulted in them being perceived more favorably as leaders and females generally felt their gender as harmful.

There are several practical implications of these results for graduate medical education. Our study suggests that programs that have been predominantly male are likely to have higher rates of implicit bias against female residents. Gender bias against female students both in and out of medicine has been previously demonstrated, though some argue that this is due to faculty representing a different generation [23, 24]. Our results indicate that implicit gender bias remains a problem among current US residents and that since there has been parity in medical student gender proportions for decades, and the current generation exhibits this bias, passive solutions to gender bias are unlikely to be successful in resolving this problem.

There is no evidence that men are rated higher and preferred as leaders because men are actually better medical team leaders compared to women. Medical teams led by women may unknowingly face challenges in efficiently providing care due to bias on the part of team members against female leaders. Teams which often include multiple residents at different training levels may be more likely to follow a male team leader as a mistake is being made, or follow a more junior or inexperienced male instead of an experienced female, as well as over-scrutinize correct decisions of female leaders. Characteristics considered more “female” may be undervalued, even when they are important attributes in patient care. Thus, implicit bias may negatively impact the ability of the healthcare system to deliver safe and efficient care. Furthermore, when safety problems arise as a result of leadership problems, female residents may be subject to greater scrutiny than their male counterparts which could lead to decreased personal confidence in themselves or decreased confidence in them as leaders by other team members [15]. A recent study demonstrated that female Emergency Medicine residents were rated lower on average than their male counterparts on the ACGME milestones by both male and female faculty members [25]. There is no evidence to suggest implicit bias is different/less in faculty members compared to residents, and implicit bias could directly contribute to the lower ratings; though implicit bias could also reduce the effectiveness of the training programs for female residents. Combined, these effects could not only negatively affect patient care, but also may contribute to the higher rates of depression observed among female physician trainees and discourage female trainees from advancing in leadership positions in their careers [26]. Residency programs should consider developing evaluation tools that systematically minimize gender bias as well as actively surveilling for gender bias in assessment of their trainees. Outside the clinical care domain, women are under-represented in leadership positions in medicine [27–29]. Lack of women in leadership positions can have a substantial impact on the emphasis of topics specific to women’s health, effectively reduces the pool of talented individuals for leadership positions, can reduce the efficacy of our training programs, and overall reduces the diversity and representativeness of leadership in medicine.

There are several potential mechanisms to reduce the potentially negative effects of implicit bias among residents and other members of the healthcare workforce including medical students and practicing physicians. First, attempts could be made to directly reduce implicit bias. Various types of interventions have shown promise in reducing implicit racial bias, though it is unclear if these types of interventions would be practical or feasible in medicine, and each intervention was relatively complex to implement [30–32]. One promising study focused on advancing female faculty careers found that a 20-min educational intervention had a small, but significant effect in reducing implicit gender bias among all participants regardless of age or gender [10]. Implicit bias awareness programs using the IAT may be considered to help reduce bias. However, it is unclear if awareness alone is sufficient to reduce implicit bias, though awareness is clearly an important first step in any organization that wishes to address implicit bias.

### Limitations

The survey we used to accompany the IAT in this analysis was adapted from a previously established survey, but in its final form is new and has not been used previously. Any new survey is subject to problems with specific questions. Next, the overall response rate for the survey was low when using all residency programs as the denominator. However, we had at least one response from 80% of residency programs indicating at least some broad level of representation of geographic sites and academic medical centers. In addition, among the core sites that participated in the sub-study, survey response rate was higher and results among this group were similar to those in the larger cohort. The IAT score results in this category were associated with low strength of bias if using the typical Cohen’s *d* strength of association convention, though the bias strength norms have not been well studied for the gender IAT and standard scores for respondents in healthcare are generally not available for comparison. In addition, statistically small IAT effects can have a profound impact on the environment due to affecting many people at once, and affecting individuals repeatedly over long periods of time [33]. It is unclear what other implicit biases may have a detrimental effect on team leadership and may contribute to patient safety problems to a greater or lesser extent than gender. Finally, there has been criticism of the IAT itself and some authors have found only small associations between the IAT and actual behavior which could influence interpretation and application of the results of the study [34].

## Conclusions

We found that gender bias is present among US residents favoring men in leadership positions, this bias differs between male and female residents, and is modified by specialty. Implicit bias does not differ across training years, and is also associated with explicit bias.

## Data Availability

The data for this study are not currently available to the public. Analysis of the raw implicit association data for this study requires a unique computer program developed specifically for this project. The raw data was collected via a website that was set up specifically for this project. Because of this, we do not believe the raw data will be meaningful or useful to readers. However, the datasets used and/or analyzed during the current study are available from the corresponding author on reasonable request.

## Abbreviations

ACGME: Accreditation Council for Graduate Medical Education
ANOVA: Analysis of Variance
CORD: Council of Residency Directors
CREOG: Council on Residency Education in Obstetrics & Gynecology
EM: Emergency Medicine
IAT: Implicit Association Test
IRB: Institutional Review Board
LLCD: Leadership Learning and Career Development Survey
OB/GYN: obstetrics and gynecology
US: United States
